# Progress and challenges in development of minimum essential dataset for disease surveillance through a One Health lens: a scoping review

**DOI:** 10.1186/s40249-026-01437-6

**Published:** 2026-04-20

**Authors:** Tianyun Li, Lijun Jia, Ne Qiang, Jinxin Zheng, Jinjun Ran, Xiaoxi Zhang, Lefei Han

**Affiliations:** 1 School of Global Health, Chinese Center for Tropical Diseases Research, Shanghai Jiao Tong University School of Medicine, Shanghai, 200025 China; 2 School of Public Health, Shanghai Jiao Tong University School of Medicine, Shanghai, 200025 China; 3 National Key Laboratory of Intelligent Tracking and Forecasting for Infectious Diseases, National Institute of Parasitic Diseases, Chinese Center for Disease Control and Prevention (Chinese Center for Tropical Diseases Research), National Health Commission of the People’s Republic of China (NHC) Key Laboratory for Parasitology and Vector Biology, World Health Organization (WHO) Collaborating Centre for Tropical Diseases, National Centre for International Research on Tropical Diseases, Shanghai, 200025 China

**Keywords:** Minimum essential dataset, Infectious disease, Surveillance, One Health

## Abstract

**Background:**

Minimum essential dataset (MED) enables One Health surveillance by facilitating cross-sectoral data sharing. Yet, existing MED research represents insufficient integration of the One Health concept, and its progress in disease surveillance remains unclear. This study aims to evaluate the current progress and gaps in MED research in disease surveillance through a One Health lens.

**Methods:**

This scoping review systematically searched Embase, PubMed, Scopus, and Web of Science from the inception of databases to November 30th, 2024 to identify studies published in English. Studies were independently screened by two reviewers for inclusion based on their relevance to the development of MEDs for disease surveillance. The progress and challenges were synthesized based on data extracted from eligible studies.

**Results:**

This review includes 28 eligible studies for analysis, all of which focused on infectious diseases. MED development is predominantly concentrated on the human health interface (89.3%), whereas animal and environmental interfaces are comparatively limited (≤ 10.7%). The One Health concept has been adopted in 16 of the eligible studies; however, there is still insufficient interdisciplinary and cross-sectoral collaboration (57.1%). Meanwhile, numerous methods have been employed in the development of MEDs, with qualitative approaches being the most prevalent (42.9%), while data-driven approaches remain scarce. The absence of a standardized approach is recognized as a primary barrier. Thus, we propose a pathway and operational tool to detail the specific steps, which could facilitate future research on MED.

**Conclusion:**

This review highlights critical gaps in MED development at the human-animal-environment interface, and proposes a pathway and operational tool to inform future development of MED, including scope identification, establishment, modification and improvement. This study promotes the improvement of disease surveillance system and provides insights to enhance future preparedness for reducing the global public health burden.

**Graphical abstract:**

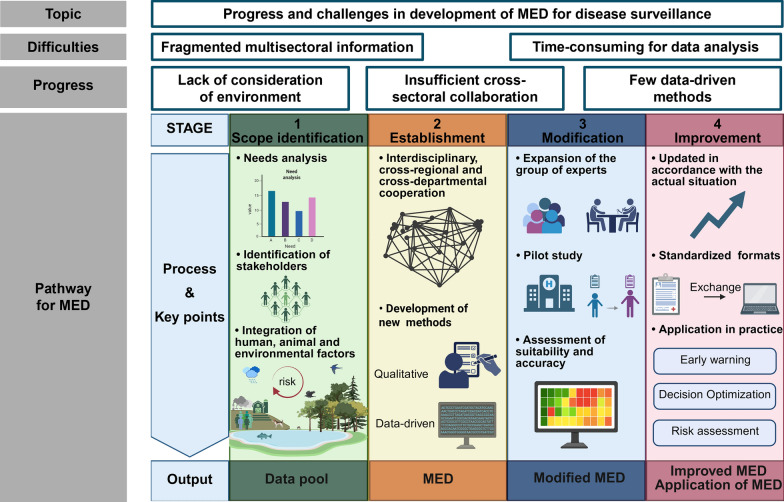

**Supplementary Information:**

The online version contains supplementary material available at 10.1186/s40249-026-01437-6.

## Background

Public health surveillance is defined as the continuous collection, analysis, and interpretation of health-related data to guide interventions [1]. As emerging infectious diseases (EIDs) become increasingly prevalent, sentinel surveillance on the human-animal-environment interface, emphasized by the concept of One Health, has gained more attention internationally [2, 3]. For example, the World Health Organization (WHO), Food and Agriculture Organization of the United Nations (FAO), World Organization for Animal Health (WOAH) and United Nations Environment Programme (UNEP) have issued the *One Health Joint Plan of Action (2022–2026) (OH JPA)* to note the importance of high-quality surveillance data to assess zoonotic disease risks [4] and the *Joint Risk Assessment Operational Tool (JRA OT)* to enhance One Health-based surveillance and mitigate pathogen spillover from animal to human [5]. However, traditional systems rely more on health institutions, such as the Centers for Disease Control and Prevention, hospitals, and public health laboratories, but less on animal and environmental institutions [3, 6].

Minimum essential dataset (MED) is critical for promoting disease surveillance based on the One Health concept. Surveillance under the One Health concept particularly focuses on disease drivers, such as land, water, climate, and wildlife [2]. The vast volume of data, independent systems [6], specific terminology [7] and heterogeneous data formats [8] across human, animal, and environment sectors impede efficient cross-sectoral data sharing [9, 10]. MED makes it possible by specifying the data collected in a standardized form [11], saving time and costs while maintaining surveillance quality, which could contribute to maximizing the benefits of disease surveillance. MED refers to a minimum set of essential data required for early detection and warning of potential infectious disease occurrences or outbreaks [12]. Its primary utility lies in integrating heterogeneous and fragmented multisectoral information into a unified surveillance framework [13, 14]. This concept still lacks a standardized nomenclature, with terms such as minimal data set, essential data set, and core data set used variably. We adopted the term MED as it aligns with the One Health framework by prioritizing essential rather than just minimal data elements. Given the multiple factors involved in addressing health threats at the human-animal-environment interface, the rational allocation of limited resources is particularly critical. Prioritizing essential data could substantially enable a timely response. Therefore, exploring the development of MEDs could facilitate One Health surveillance, which in turn contributes to the improvement of public health.

So far, numerous institutions and countries have taken actions to strengthen disease surveillance systems [15, 16]. Recognizing MED as a pivotal tool for it, relevant research on the development of MEDs has garnered increasing attention. For instance, a working group of WHO and European Region of the International Union Against Tuberculosis and Lung Disease has jointly developed a MED for tuberculosis surveillance across European countries, which allows cross-country epidemiological comparisons [17]. Haghiri et al. established a MED through a comprehensive review of notifiable disease surveillance systems, thereby advancing global standardization in notifiable disease reporting and management [18]. Nationally, Iran has established a MED for coronavirus disease 2019 (COVID-19) to strengthen surveillance [13]. China established MEDs for tuberculosis and major diseases [19, 20]. However, those MEDs do not sufficiently integrate the human-animal-environment interface, resulting in the absence of some essential monitoring data. To our knowledge, there were no studies that have systematically reviewed research on MED from a One Health perspective to support the improvement of disease surveillance system.

Therefore, we performed a scoping review to systematically synthesize studies on MED relevant to disease surveillance through the One Health lens. This study aims to evaluate the current progress, identify research gaps, and propose future research priorities, which may advance the application of MED to strengthen the surveillance system.

## Methods

This study was guided by the methodological framework developed by Arksey and O’Malley [21], and the Preferred Reporting Items for Systematic Reviews and Meta-Analyses extension for Scoping Reviews (PRISMA-ScR) checklist and explanation developed by Tricco et al. [22].

### Development of search strategy

Embase, PubMed, Scopus, and Web of Science were searched for studies published in English from the inception of the database to November 30th, 2024. Terms for minimum essential dataset (e.g., “minimal dataset” OR “minimal data set” OR “minimal database” OR “minimum dataset” OR “minimum data set” OR “minimum database” OR “essential dataset” OR “essential data set” OR “essential database” OR “core dataset” OR “core data set” OR “core database”), surveillance (e.g., “surveillance” OR “monitoring”), and report (e.g., “reporting” OR “report” OR “notification”) were combined with boolean logic (“AND”, “OR”) to construct search strategies (Additional file: Table S1). In addition, Google Scholar and references of eligible studies were searched to supplement and refine the results.

### Inclusion and exclusion criteria

Prior to the formal screening, fifty studies were randomly selected for initial screening. These studies were independently evaluated by two reviewers (TL, LJ) to calibrate inclusion and exclusion criteria [23]. Any disagreements were discussed and resolved in consultation with our research team. The screening of eligible studies was guided by the following inclusion criteria: (i) studies that establish MEDs or propose the need to establish MEDs for disease surveillance; (ii) studies involving diseases or elements used in surveillance with a potential to spread or circulate across the human-animal-environment interface; (iii) studies in English; (iv) studies that have been peer-reviewed. The following literature was excluded: (i) studies that apply previously established MEDs for analysis without contributing to their development; (ii) studies focusing on diseases outside the human-animal-environment interface; (iii) studies whose full text is not available.

### Studies screening

Studies obtained from all databases were imported into the software EndNote 21 (Clarivate, Philadelphia, USA) for analysis. The screening was performed strictly following inclusion and exclusion criteria in a two-step process: first, an initial screening was conducted based on titles and abstracts; next, articles for final inclusion were identified by reading the full text. Screening of studies was carried out independently by two reviewers (TL, LJ) with the timely discussion of differences. A third reviewer (LH) was invited to participate in the discussion of disagreements until agreement was reached.

### Quality assessment of included studies and data extraction

A descriptive quality assessment was conducted to evaluate the methodological rigor, relevance to MED construction, and clarity of reporting of each included study. This assessment guided the synthesis and interpretation of findings. Extraction of key information was carried out independently by two reviewers (TL, LJ), checked in two rounds with the support of a third reviewer (LH), and finally agreed upon by our research team. The following information was extracted to provide a comprehensive understanding of research advances in MED related to disease surveillance: year, title, disease, territorial dimension, establishment of MED (yes or no), methods for the establishment of MED, subject of MED, quantity of elements in MED, elements in detail/summary of MED, and recommendations.

## Results

### Summary of eligible studies

**Fig. 1:**
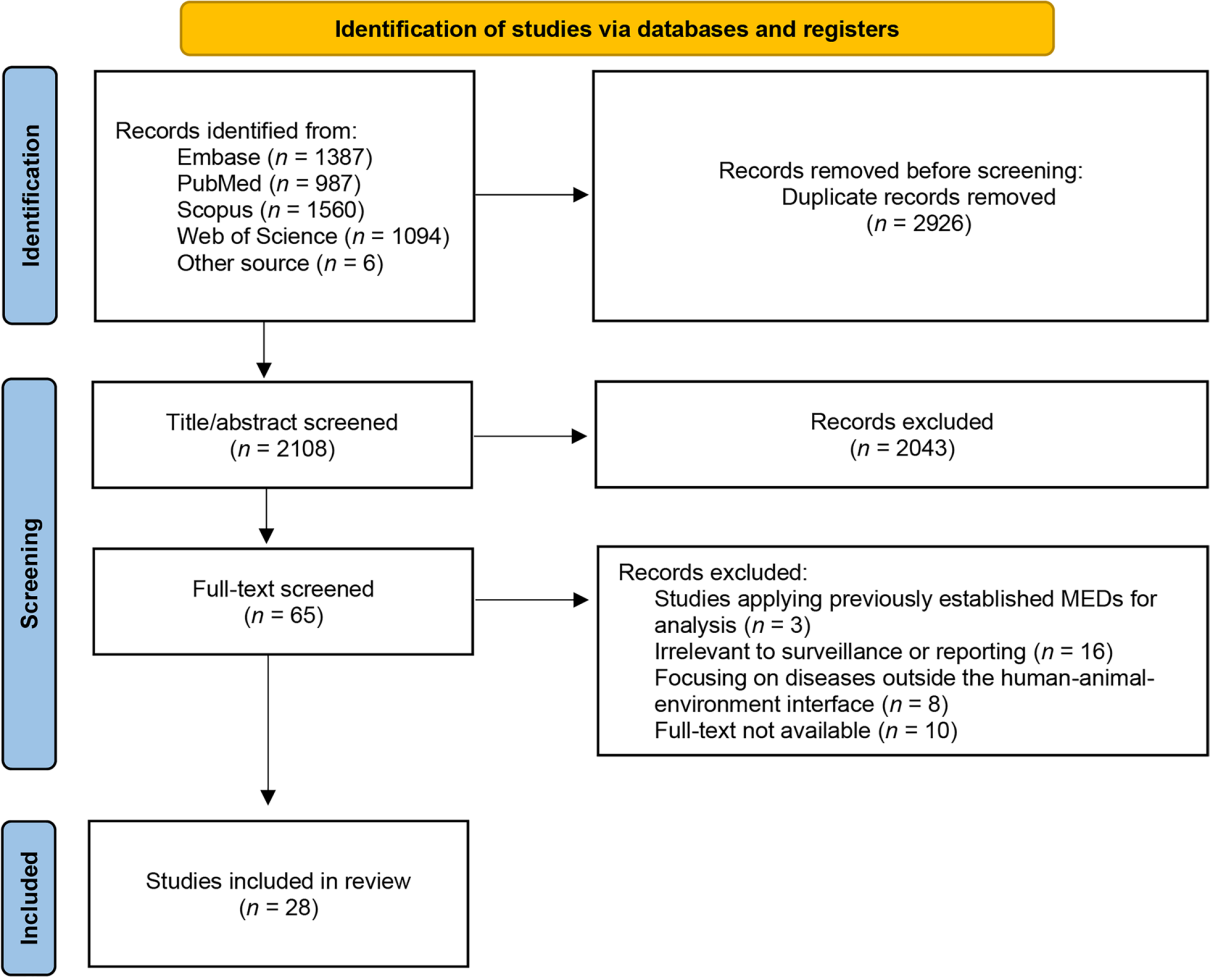
Flow chart depicting the screening and inclusion process. Other sources: Google Scholar and references of eligible literature. Abbreviation: MED, minimum essential dataset

A total of 2108 studies were initially identified after removing duplicates, including 2102 from electronic databases and 6 from other sources. Of these, 2043 were excluded as irrelevant, and the full text of the remaining 65 studies were assessed for eligibility. A further 37 studies were excluded, including those based on existing MEDs for analysis (*n* = 3), those not relevant to surveillance or reporting (*n* = 16), those focusing on diseases outside the human–animal–environment interface (*n* = 8), and those without available full text (*n* = 10). Ultimately, this review included 28 eligible studies. The screening and inclusion process is shown in Fig. 1.

### Characteristics of eligible studies

All eligible studies focused on infectious diseases, and their information is summarized in Table 1. In terms of content, twelve studies reported developing process of MED [6, 8, 13, 18, 24–31], eight studies summarized the previously established MEDs [17, 32–38], and eight studies provided recommendations on the MED development [39–46]. The main content and implementation process of eligible studies are summarized in Additional file: Table S2. We synthesized the extracted information from four key perspectives: scope of MED, establishment of MED, modification of MED, and improvement and application of MED.

**Table 1:** Basic information of eligible studies

Year	Title	Disease	Territorial dimension	Establishment of MED	Methods for the establishment of MED	Subject	Quantity of elements in MED	Elements in detail/ Summary of MED	Recommendations
2022	Common data elements and features of brucellosis health information management system [24]	Brucellosis	Asia	Yes	Literature review and 2 round Delphi survey	Human	134	Administrative information (14), epidemiology (7), diagnosis investigation (35), complications (26), and signs and symptoms (52)	Further extensions and modifications; Conduct an experimental study by including a supplementary Delphi phase to improve the dataset; Assess this MED from the standpoints of a greater panel of experts
2020	Coronavirus disease 2019 (COVID-19) surveillance system: Development of COVID-19 minimum data set and interoperable reporting framework [13]	COVID-19	Asia	Yes	Literature review and expert consensus	Human	69	Administrative data (29), clinical data (40)	The development of conceptual models of surveillance systems; Conducting a pilot study including a further Delphi stage prior to refine some data categories; Access this MED from the perspectives of a greater group of clinical and public health professionals
2020	Design and development of a web‐based registry for COVID‐19 [25]	COVID-19	Asia	Yes	Literature review and 2 round Delphi survey	Human	56	Administrative part (30), and the clinical part (26)	Further development and adjustments of this MED; Conducting a pilot study, including a further Delphi step to refine the MED; This MED may need to be evaluated from the perspectives of larger group of medical and public health experts to be applicable at the national level; The Delphi consensus approach has its restrictions is that most views are marginalized
2022	Designing a standardized framework for data integration between zoonotic diseases systems: Towards one health surveillance [8]	Zoonotic diseases	Asia	Yes	Literature review and 2 round Delphi survey	Human, animal and environment	95	Administrative data (38), and clinical data (57)	This MED might need to be assessed from the standpoints of a larger assembly of medical and public health experts to be valid nationwide; Developing conceptual models of surveillance systems and conducting a pilot study including a further Delphi stage before refining some data categories
2020	Development of minimal basic data set to report COVID-19 [26]	COVID-19	Asia	Yes	Data collection and expert panel	Human	125	Epidemiological data (22), clinical data (57) and paraclinical data (46)	Future testing in other health care settings; A comprehensive search of the literature to enhance this MED
2017	Identification of the necessary data elements to report AIDS: a systematic review [28]	AIDS	Asia	Yes	Systematic review	Human	205	Administrative, managerial, and policy-makers (90), Clinical and medical (83), and support services, counselling and subsidiary (32)	–
2021	Notifiable diseases interoperable framework toward improving Iran public health surveillance system: lessons learned from COVID-19 pandemic [29]	Infectious diseases	Asia	Yes	Literature review and 2 round Delphi survey	Human	96	Clinical data (57), and nonclinical data (39)	The urgency of reporting some notifiable diseases is likely to be changed due to mandatory report of new conditions or even excluded some diseases from the present list, which may necessitate modifications to the MED in the future
2019	Notifiable diseases surveillance system with a data architecture approach: a systematic review [18]	Infectious diseases	North America, Europe, Asia, Africa, and Oceania	Yes	Systematic review	Human	77	Clinical data (38) and nonclinical data (39)	It is recommended that a list of core data elements be provided for notifiable diseases to be used for reporting at local and national levels; In order to create a MED for public health purposes, the special needs (specific case reporting) should be considered with patient identification information at local levels, from case detector organizations to local public health organizations; The most important data quality criteria were completeness, accuracy, and timeliness of data
2021	Regional COVID-19 registry in Khuzestan, Iran: A study protocol and lessons learned from a pilot implementation [30]	COVID-19	Asia	Yes	Literature review, expert panel discussion sessions and pilot study	Human	396 (45 are compulsory)	‏Administrative data (39), disease and encounter data (65), medical history and physical examination (121), findings of clinical diagnostic tests (95), disease progression and outcome of treatment (39), medical diagnosis and cause of death (14), and follow-up data (23)	–
2021	A study to design minimum data set of COVID-19 registry system [31]	COVID-19	Asia	Yes	Literature review, expert panel discussion sessions and pilot study	Human	434	Administrative (34), disease exposure (61), medical history and physical examination (138), findings of clinical diagnostic tests (101), disease progress and outcome of treatment (55), medical diagnosis and cause of death (12), follow-up (14), and COVID-19 vaccination (19) data	Strongly recommending an international MED be designed for the long-term complications of COVID-19
2008	Web-based infectious disease reporting using XML forms [6]	Infectious diseases	Asia	Yes	Data collection and expert panel	Human	23	4 sections: patient identifier, patient contact information, problems of the patient, pesponsible organization/personnel identifying information	–
2001	The EUVAC-NET survey: national pertussis surveillance systems in the European Union, Switzerland, Norway, and Iceland [27]	Pertussis	Europe	Yes	Qualitative study (Questionnaire)	Human	8	Age, gender, area of residence, date of disease onset, vaccination status, admission to hospital, death as disease outcome, case classification (suspected/confirmed)	–
2010	Effective animal health disease surveillance using a network-enabled approach [33]	Animal disease	North America	No (summarize previously established MDSs)	–	Human and animal	–	This group has allowed for significant inter-jurisdictional co-operation in developing techniques and tools for predicting, detecting, analyzing and controlling disease including the "Minimum Data Set". This data set has achieved a standardized, core minimum data set of surveillance information that can be used for any animal disease surveillance programme	–
2021	Response measures to COVID-19 in prisons and other detention centers [35]	COVID-19	Globe (Prisons)	No (summarize previously established MDSs)	–	Human	–	–	–
2022	The World Health Organization COVID-19 surveillance database [38]	COVID-19	Globe	No (summarize previously established MDSs)	–	Human	–	WHO has established MDS to report COVID-19	As the disease spread and numbers of cases increased, the burden on surveillance systems impacted the capacity to provide detailed case-based data, and in March 2020, recommendations for surveillance data reporting shifted to weekly aggregate reporting of a minimum global dataset comprising age and sex disaggregation of cases and deaths, newly hospitalized cases, and occupation to capture cases among Health Care Workers (HCW)
1996	Surveillance of tuberculosis in Europe. Working Group of the World Health Organization (WHO) and the European Region of the International Union Against Tuberculosis and Lung Disease (IUATLD) for uniform reporting on tuberculosis cases [17]	Tuberculosis	Europe	No (summarize previously established MDSs)	–	Human	5	Date of starting treatment, place of residence, date of birth, gender, and country of origin	The minimum set of variables should be collated on all patients and should be as complete as possible. Additional variables may be collected for individual, local or national purposes, but, in general, completeness of reporting on cases is likely to be better if the information requested is kept to a minimum
2007	A surveillance network for meningococcal disease in Europe [37]	Meningococcal disease	Europe	No (summarize previously established MDSs)	–	Human	8	The agreed minimum data set comprises information on age, sex, date of onset, method of laboratory confirmation, site of identification, serogroup, serotype and serosubtype	–
2023	Architecture assessment of the Chilean Epidemiological Surveillance System for Notifiable Diseases (EPIVIGILA): qualitative study [32]	Infectious diseases	Europe, Oceania, North America, Asia, and Africa	No (summarize and compare previously established MDSs)	–	Human	–	–	–
2017	National Communicable Disease Surveillance System: A review on information and organizational structures in developed countries [34]	Infectious diseases	North America, Oceania and Europe	No (summarize and compare previously established MDSs)	–	Human	–	Demographic data, laboratory, clinical and vaccination data	–
2019	Standardising surveillance of hepatitis E virus infection in the EU/EEA: A review of national practices and suggestions for the way forward [36]	Hepatitis E virus infection	Europe	No (summarize and compare previously established MDSs)	–	Human	–	Unique patient identifier, date of notification, source of notification, date of birth/age, sex, date of onset of disease	A MED describing the epidemiology of laboratory-confirmed cases was suggested to include date of diagnosis, age, sex and place of residence
2014	Accuracy of epidemiological inferences based on publicly available information: retrospective comparative analysis of line lists of human cases infected with influenza A(H7N9) in China [39]	Influenza A (H7N9)	Asia	No (provide recommendation)	–	Human	–	–	A MED with standardized format and definition
2007	Duration and distance of exposure are important predictors of transmission among community contacts of Ontario SARS cases [40]	Severe acute respiratory syndrome (SARS)	North America	No (provide recommendation)	–	Human	–	–	Separate measures of time and distance from source cases should be added to MED for the assessment of interventions for SARS and other emerging diseases
2013	Elimination of tropical disease through surveillance and response [41]	Neglected tropical diseases (NTDs)	Globe	No (provide recommendation)	–	Human	–	–	Establish MED for the elimination of tropical diseases
2004	Methodological and quality issues in epidemiological studies of acute lower respiratory infections in children in developing countries [42]	Acute lower respiratory infections (ALRI)	Developing country	No (provide recommendation)	–	Human	–	–	Establish MED for acute lower respiratory infections
2015	A practical approach to designing syndromic surveillance systems for livestock and poultry [43]	Animal syndromic	North America	No (provide recommendation)	–	Animal	–	–	A MED for animal syndromic surveillance that includes the essential data to achieve all surveillance objectives while minimizing the amount of data collected should be defined
2004	Surveillance systems for STIs in the European Union: Facing a changing epidemiology [45]	Sexually transmitted infections (STIs)	Europe	No (provide recommendation)	–	Human	–	–	Definition of standardized minimum datasets is needed for STI
1996	Surveillance of infectious diseases in the European Union [44]	Infectious diseases	Europe	No (provide recommendation)	–	Human	–	–	It would be better to decide on the MED to be shared across countries for the important diseases before we embark on detailed epidemiological investigations of specific infections
2020	Transmission dynamics: Data sharing in the COVID-19 era [46]	COVID-19	North America	No (provide recommendation)	–	Human	–	–	Determine a MED; New data elements may be needed to supplement the prespecified minimum data set in order to explore these and other future analysis

*MED* minimum essential dataset

#### Scope of MED

All the eligible studies identified the theme focus and interface. In terms of theme focus, twelve studies developed MEDs focused on disease surveillance [6, 8, 13, 18, 24–31], with four additionally focusing on disease reporting [6, 24, 26, 28]. There were 25 (89.3%) studies that focused primarily on human health information, in which the MEDs were mainly developed for notifiable infectious diseases (e.g., tuberculosis and brucellosis) or emerging infectious diseases [e.g., COVID-19 and influenza A (H7N9)] and comprising core data elements such as onset date, reporting date, location of exposure, travel history, and clinical symptoms [6, 13, 17, 18, 24–32, 34–42, 44–46]. Two studies (7.1%) reported developing MEDs for animal diseases and animal syndromes [33, 43]. They highlight the value of animal disease surveillance in preventing pathogen transmission from animals to humans, and primarily focused on data elements of MEDs related to the animal interface (e.g., species, livestock density, mortality rate). Only one study (3.6%) established a MED for zoonotic diseases at the human-animal-environment interface [8]. This study integrated human factors (e.g., exposure history, transmission route), animal factors (e.g., farming**/**husbandry approaches, wildlife biodiversity), and environmental factors (e.g., cultivation rate, desert, rangelands, rainy forest, climate) (Additional file: Fig. S1A).

In terms of geographic scope, most of these studies were conducted at the national level (42.9%) [6, 8, 13, 24–26, 28, 29, 33, 39, 43, 46], while few studies were conducted at the sub-national level (10.7%) [30, 31, 40] (Additional file: Fig. S1B). Among all regions, Asia accounted for the largest proportions of MED-related studies, with 13 (46.4%) studies identified [6, 8, 13, 18, 24–26, 28–32, 39]. Moreover, the established MEDs were mostly in middle socio-demographic index (SDI) regions, with a few in low- and middle-low SDI regions [6, 8, 13, 18, 24–31].

We evaluated the alignment of the included studies with the OH JPA (Additional file: Fig. S1C) and mapped their objectives and associated diseases with the OH JPA’s action tracks to assess research progress (Additional file: Table S3). Generally, a total of 16 studies (57.1%) aligned with the OH JPA’s action tracks. For action track 1, there were three studies that recommended enhancing MED by strengthening cross-regional risk surveillance and integrating human and animal surveillance data [8, 33, 41]. For action track 2, there were 13 studies that focused on emerging and re-emerging infectious diseases, such as brucellosis, COVID-19, severe acute respiratory syndrome (SARS), and influenza A (H7N9) [8, 13, 17, 24–26, 30, 31, 35, 38–40, 46]. These studies involved collaboration among cross-sectoral and interdisciplinary experts, embodying the joint prevention and control strategy. For action track 3, there were two studies focusing on MEDs for zoonotic diseases [8] and neglected tropical diseases (e.g., schistosomiasis and visceral leishmaniasis) [41], specifically considering data at the human-animal-environment interface. For action track 4, one study established a MED for hepatitis E virus infection, highlighting its role in supporting risk assessment and management in food safety [36]. For action track 6, one study integrated environmental risk factors into a MED for zoonotic diseases, such as climate change and geography characteristics [8]. No studies were identified that aligned with action track 5.

#### Establishment of MED

The establishment of the MED required intersectoral and interdisciplinary collaboration. In the process of screening the indicators to establish the MED, the cross-sectoral collaborators were mainly from public health organizations, hospitals and animal health centers. Multidisciplinary experts have participated in discussions, such as clinical medicine and veterinary medicine [6, 8, 13, 18, 24–31]. In addition, the indicator screening methods relied exclusively on qualitative approaches, with multiple methods typically combined for implementation (Additional file: Table S2). There were five studies combining literature search and Delphi survey [8, 13, 24, 25, 29]; two studies combining data collection and expert panel [6, 26]; and two studies combining literature review, expert panel discussion sessions and pilot study [30, 31]. Moreover, individual methods, including systematic review [18, 28] and qualitative study (using questionnaire) [27], were adopted in three studies (Additional file: Fig. S1D). Few data-driven approaches have been applied in this field. Moreover, MEDs developed at larger geographic scales typically employed simpler methods with fewer data elements, due to greater regional heterogeneity [17, 27, 30, 31, 37]. In contrast, the refinement and applicability of MED increased as the scope of the study was narrowed.

#### Modification of MED

Expanded expert groups and pilot studies were common approaches to validate and modify MED. Four studies recommended that the elements included in MED should be further assessed from the perspectives of a greater group of experts, thus reducing the subjective influence [8, 13, 24, 25]. Two studies mentioned that pilot studies could test the applicability of MEDs and guide modification [8, 13], which were usually conducted in hospitals to collect the information of patients on-site. The availability of data elements in the MEDs was verified, and the elements were added or removed as appropriate. Simultaneously, the suitability and accuracy of MED could be assessed during this stage.

#### Improvement and application of MED

Since the characteristics of disease transmission and epidemiology were constantly changing, seven studies recommended that the established MEDs should be kept up-to-date [17, 24–26, 29, 40, 46], which could be better adapted to the current surveillance work. In addition, four studies noted that the modified MEDs required a matching collaboration platform [18, 24, 33, 43], and the standardization of data formats was key to the smooth functioning of the platform. Recommendations provided in eligible studies and their explanations are summarized in Table 2.

**Table 2:** Recommendations for future research on MED

Gaps	Recommendations	Explanation	References
Currently used methods are qualitative and lack of data-driven methods	Further assessment of MED from the perspectives of a greater group of experts to reduce subjective bias in the use of qualitative methods	About 30 experts are usually selected for discussion when establishing a MED, which will subject the results of the study to the subjective influence of a small group of experts. Therefore, inviting more experts to assess the results could reduce supervisory bias to some extent. It is recommended that a panel of 100 or more experts should be selected	Shafiee et al. [24]; Shanbehzadeh et al. [13]; Kazemi-Arpanahi et al. [25]; Shanbehzadeh et al. [8]
Established MED are not up-to-date	Update and supple the MED	The applicability of MED changes with the actual situation, e.g., pathogen variation, mode of disease transmission, etc. The original data elements in the MED need to be adapted to the actual situation. For example, if a study proves that A is an important risk factor, the element A can be added to the MED. Factors that have less impact on the current situation could be considered for deletion	Shafiee et al. [24]; Kazemi-Arpanahi et al. [25]; Shanbehzadeh et al. [26]; Shanbehzadeh et al. [29]; Rieder et al. [17]; Rea et al. [40]; Foraker et al. [46]
Most established MED remain at the theoretical level without practical validation	Pilot study is important to modify MED	Pilot studies are usually chosen to be conducted in hospitals where all information in MED is collected on-site. This process enables the identification of elements that must be collected and the addition of new elements that are found to be necessary during practice	Shanbehzadeh et al. [13]; Shanbehzadeh et al. [8]
Technical barriers to the practical extension of the use of established MED	Standardized MED are needed	In order to increase the possibilities and efficiency of data exchange, standards are set for the data collected by each agency or department. Data collected according to this standard is harmonized and may facilitate data integration	Haghiri et al. [18]; Zarei et al. [31]; Allan et al. [38]; Lau et al. [39]; Zhou et al. [41]; Lanata et al. [42]; Vial et al. [43]; Lowndes et al. [45]; Giesecke [44]; Foraker et al. [46]

*MED* minimum essential dataset

### Gap analysis

**Fig. 2:**
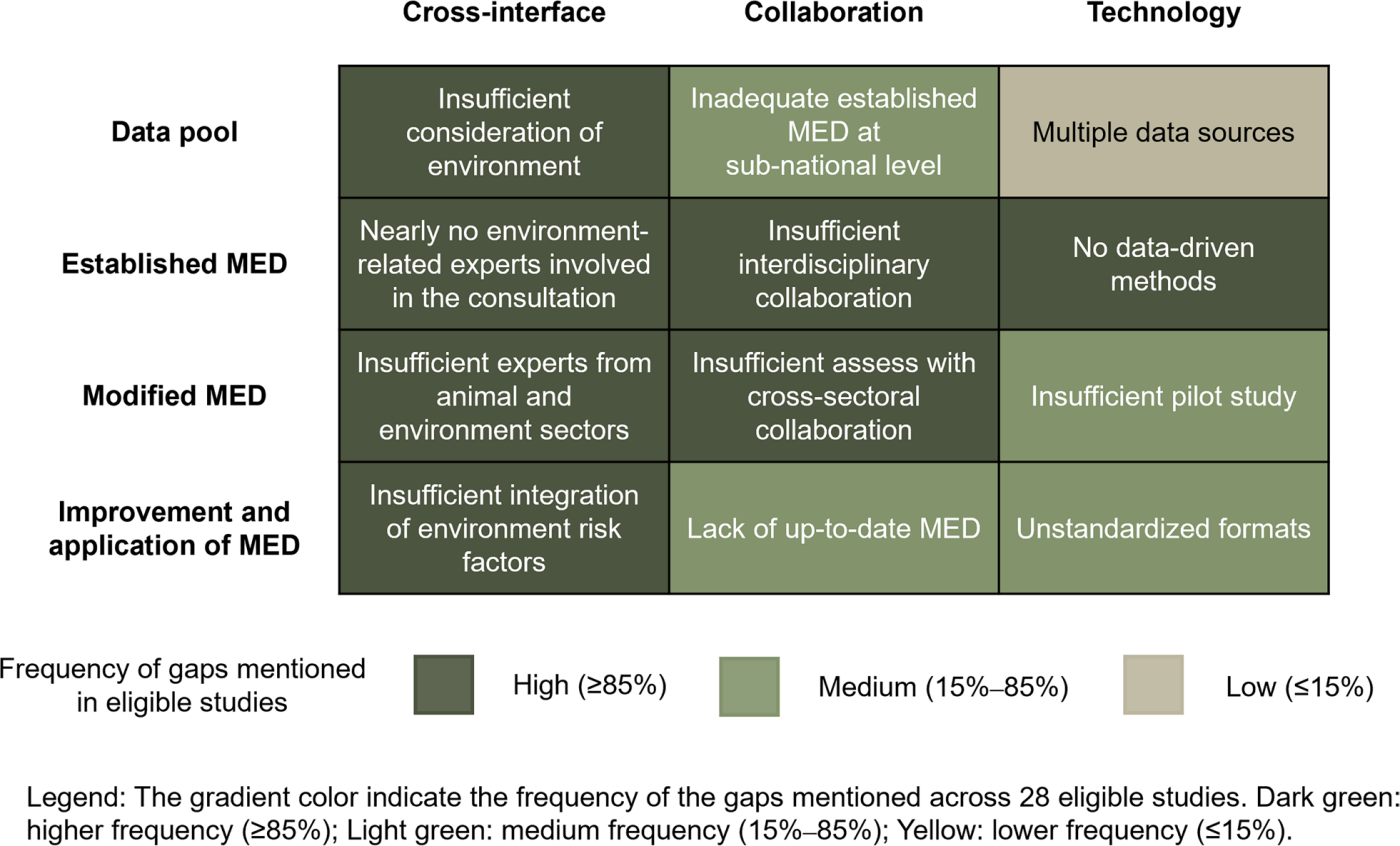
Gap analysis for minimum essential dataset. Abbreviation: MED, minimum essential dataset

A heat map showed the research gaps derived from eligible studies. The challenges were analyzed in terms of cross-interface, collaboration, and technology, which were ranked according to their importance and urgency (Fig. 2).

#### Insufficient cross-interfaces across humans, animals, and the environment

Despite great attention given to zoonotic diseases, studies were mainly focused on human health issues, with few animal-related data elements included in MEDs established at the human-animal interface [8, 13, 17, 24–26, 30, 31, 35, 38–40, 46]. In addition, most studies inadequately addressed environmental risks [6, 8, 13, 18, 24–31]. This gap indicates that environmental determinants were insufficiently integrated into MEDs, thereby limiting their utility for comprehensive risk assessment and informed decision-making. It also reflected that existing MED studies did not fully align with the One Health framework that emphasizes integrated monitoring across human-animal-environment interfaces.

#### Insufficient interdisciplinary, cross-regional and cross-sectoral collaboration

The process of developing the MED did not fully put into practice the multisectoral and multidisciplinary collaboration emphasized by the One Health concept. In terms of geographic regions, few were at the sub-national level [30, 31, 40], suggesting that researchers were not paying enough attention to smaller geographic areas. Therefore, collaboration between different sub-national regions was limited, posing a challenge for delicate management of MED. In addition, the establishment of the MED involved cooperation mainly restricted to public health sectors and medical institutions, leaving out the animal health and environmental health sectors [6, 8, 13, 18, 24–31]. Lack of cross-sectoral collaboration led to a lack of integration of resources from multiple sectors when establishing, modifying, and validating MED, with a consequent reduction in the applicability of the results. Moreover, a single sector was unable to identify problems in time when the MED was actually applied, which affected the improvement of the MED in keeping up-to-date.

#### Technical barriers

The lack of appropriate methods and tools was an important technical barrier to the development of MED. To date, there were few studies establishing a MED using data-driven methods [6, 8, 13, 18, 24–31]. Qualitative methods made the results more subjective to researchers and experts [24]. In addition, the inadequacy of the pilot studies [8, 13] resulted in the establishment of a MED that remained at the theoretical level and could not be applied in practice. Finally, only two studies have established a matching data exchange platform for the application of MEDs [25, 30], and five studies have standardized the data format when developing MEDs [6, 8, 13, 29, 30], which hinder the application of MED.

## Discussion

This scoping review summarized the current progress of the MED established for disease surveillance, identified research gaps and explored further research directions. Our results indicated that infectious diseases were the focus of research attention. While the One Health concept has been applied in part of the eligible studies, interdisciplinary and cross-sectoral collaboration remained insufficient. Human diseases received a great deal of attention, yet there was inadequate focus on the human-animal-environmental interface. The methods employed for MED were predominantly qualitative, with few applications of data-driven approaches reported. To date, a standardized methodology has not yet been developed, which was considered the key barrier. It is recommended to develop a pathway to detail the specific steps and operational tool, which could facilitate research on MED.

**Fig. 3:**
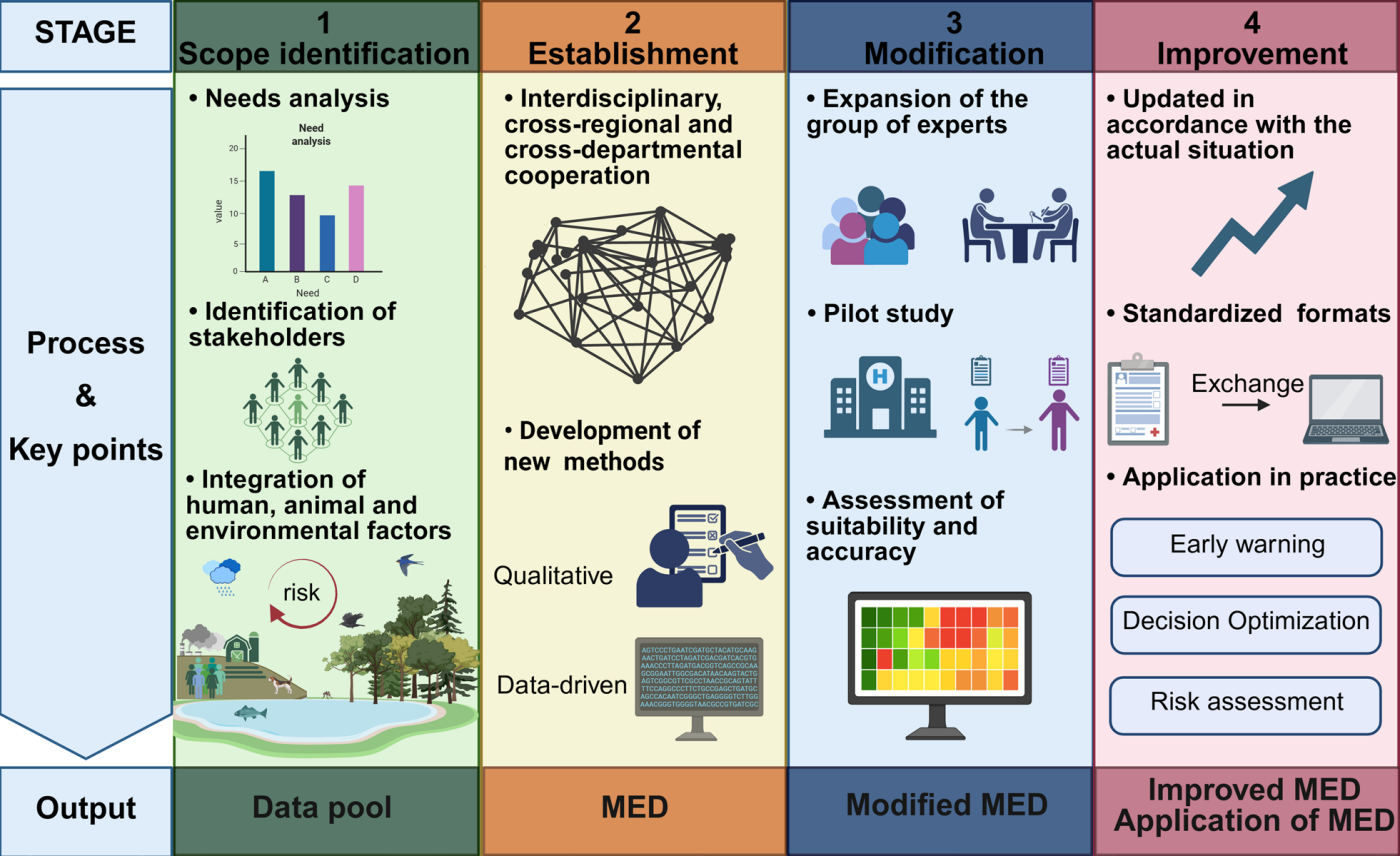
A potential pathway for the development of minimum essential dataset. Abbreviation: *MED* minimum essential dataset

Based on the eligible studies, we recommended that future research about MED could be explored based on the following four steps: (i) Scope identification: synthesizing factors across human-animal-environment interface to obtain a data pool; (ii) Establishment: screening indicators by Delphi or data-driven methods to establish a MED; (iii) Modification: validating, modifying, and assessing the established MED in the real-world contexts to develop a modified MED. This is considered pivotal for translating MED from theory to practice; and (iv) Improvement: putting the modified MED into practice and improving it (Fig. 3). The operational tool provided description, methods and implementation tips for developing MED, along with specific operational recommendations. The content includes scope of MED, establishment of data pool, establishment of MED, validation of MED, application of MED, and updates to MED (Table 3, Additional file: Fig. S2).

**Table 3:** Operational tool for developing MED

Process	Content	Description	Methods	Implementation tips
1	Scope of MED	Determine the geographic scope, disease categories, and the interfaces of focus (involving humans, animals, or the environment) for the study based on research progress and practical needs	–	Geographic scope: Determine whether the study will be conducted at the national, regional, or global level, depending on the disease’s impact and the researchers’ focus Disease categories: Typically, diseases are selected based on their high-risk characteristics—such as rapid transmission and severe health consequences—within a defined geographic area Interface focus: Applying the One Health approach to establish a MED requires identifying the specific interface (human-animal-environment) where the disease operates. This helps pinpoint relevant risk factors and stakeholders. For example, brucellosis exists at the human-animal interface, so MED should incorporate risk factors related to both humans and animals. Additionally, experts and researchers in both human and animal health should be involved in the study
2	Establishment of data pool	Extract all required data elements for monitoring the target diseases from existing materials	Literature review/Data collection	Literature review: Conduct a literature search using a predefined set of keywords, and systematically review the findings to comprehensively integrate all required data elements Data collection: Searching information from literature, information systems, report, document and etc This process should incorporate all relevant factors across the research interface. For example, dengue fever exists at the human-animal-environment interface, requiring comprehensive consideration of humans, mosquito vectors, and meteorological factors
3	Establishment of MED (screening data elements)	Based on the data pool, identify and select the subset of data that significantly impacts expected outcomes (e.g., disease incidence) to form the MED	Two-round Delphi/Data-driven methods	Two-round Delphi: Guided by the interdisciplinary and cross-sectoral approach, engage researchers and experts from multiple disciplines and sectors in the indicator selection process. The specific disciplines and sectors involved should be determined by the research interface under study Data-driven methods: Selecting indicators based on inherent data patterns is considered a viable approach. However, this method has not yet been applied within this research field. Future studies should actively explore feasible data-driven methodologies to address this gap
4	Validation of MED	Validate the initially established MED through field application, then refine it by addressing any identified limitations to produce the final version	Pilot study	Existing pilot studies are mainly conducted in hospitals, with trained personnel collecting data on-site. These studies could assess the applicability of the MED in real-world settings, including data accessibility and optimal data collection methods. Based on participants’ feedback and suggestions regarding the MED’s data elements, modifications are made, such as adding or removing data elements. The selection of sites should be expanded when validating the MED based on the One Health concept, focusing on sites at the human-animal-environment interface, such as farms
5	Application of MED	Utilize the established MED to conduct research and address complex public health challenges	Early warning/Risk assessment/Decision optimization	Early warning/Risk assessment: When the criteria for selecting data elements in the MED are based on disease burden indicators, these elements serve as key predictors of disease risk. Conducting risk analysis using the MED not only offers better cost-effectiveness but also improves analytical efficiency, enabling faster responses in health policy Decision optimization: The data elements in the MED serve as key control measures for preventing adverse outcomes. Using the MED to optimize health decision-making helps keep critical risk factors within manageable limits, reducing the impact of health threats
6	Updates to MED	Adjust the data elements in the MED based on actual conditions	–	The transmission patterns of diseases, post-infection symptoms, and scope of impact might evolve over time—typically observed in real-world settings. Additionally, the risk factors requiring attention vary across different scenarios. Therefore, the MED should fully account for both the dynamic nature of diseases and shifting analytical contexts, making adjustments based on current real-world conditions

*MED* minimum essential dataset

One Health approach should be applied to improve the stage of identification of scope. Needs analysis determined the geographic scope of the study, during which cross-regional cooperation should be considered. Smaller study areas, such as the sub-national level, were recommended to conduct more refined studies. In addition, the identification of stakeholders determines the interface involved in the research. Existing disease surveillance systems primarily collect human-related data, with inadequate consideration of animal and environmental risk factors [3]. Notably, the environment was recognized as an important reservoir of infectious pathogens, e.g., sewage-related pathogen transmission [47]. Establishment of MED based on One Health concept was recommended to incorporate animal and environmental data elements [48].

When developing a MED, the screening of indicators based on the One Health concept should take full account of interdisciplinary and cross-sectoral cooperation. Experts from human, animal, and environment fields should be involved in the discussion, enabling knowledge integration and promoting the comprehensiveness of the included data elements [3]. Moreover, updating the methodology for establishing MED was important, which could effectively improve the quality of MED. Existing studies were limited to qualitative methods, which were susceptible to subjective opinions that could bias results. The development of data-driven methods will provide additional opportunities for the establishment, validation and revision of MED [49]. Previous studies have shown that machine learning performed well in public health surveillance [50] and risk assessment [51]. Accordingly, integrating such approaches with MEDs could be a feasible strategy to enhance disease monitoring capabilities.

The modification and application of MED require a robust information-sharing platform. Well-established MEDs are important for risk assessment, early warning and policy analysis [41]. Such a platform could facilitate the application of MED in disease surveillance through the collection, integration and analysis of data [33]. Systematic integration of data plays a key role in identifying monitoring gaps and modifying MED. The construction of information-sharing platforms faces great challenges, such as inadequate support from national policies, laws and regulations [3, 16]. It is recommended that each country clearly stipulates the participating sectors and key steps in the construction of the information-sharing platform in its policies. Simultaneously, regular monitoring of work progress and result validation should be implemented to ensure the stability of the platform.

There were several limitations in this study. First, only English literature was included in this study, which may have resulted in the loss of some insights related to the topic published in other languages. Second, only four databases, Embase, PubMed, Scopus and Web of Science, were systematically searched and future studies should expand the search to include results from more databases. Third, the review only qualitatively analyzed the content of the included literature and lacked quantitative data analysis. Future studies will incorporate assessment of article quality and quantitative analysis to dig deeper into the topic.

## Conclusion

This review systematically summarizes research on MED for disease surveillance, providing insights into current progress and remaining gaps. The proposed pathway and operational tool could guide the integration of human, animal and environmental factors and standardize the process of developing MED, facilitating the development and application of MED through lens of One Health. The study provides important evidence to enhance One Health capacities to strengthen disease surveillance systems and reduce the global public health burden.

## Supplementary Information

Additional file 1.

## Data Availability

No datasets were generated or analysed during the current study.

## Abbreviations

COVID-19: Coronavirus disease 2019
EIDs: Emerging infectious diseases
FAO: Food and agriculture organization of the United Nations
JRA OT: Joint risk assessment operational tool
MED: Minimum essential dataset
OH JPA: One Health joint plan of action (2022–2026)
PRISMA-ScR: Preferred Reporting Items for Systematic Rreviews and Meta-analyses extension for scoping reviews
SARS: Severe acute respiratory syndrome
SDI: Socio-demographic index
UNEP: United Nations environment programme
WHO: World health organization
WOAH: World organization for animal health
