# Supplementary material for: Progress and challenges in development of minimum essential dataset for disease surveillance through a One Health lens: a scoping review
Source: Infect Dis Poverty. 2026 Apr 20;15:45. doi: 10.1186/s40249-026-01437-6 (PMC13093924; doi:10.1186/s40249-026-01437-6)
Supplement: Supplementary file 1 — Additional file 1. [file 40249_2026_1437_MOESM1_ESM.docx]

Additional file

**Progress and challenges in development of minimum essential dataset for disease surveillance through a One Health lens: a scoping review**

Content

[Table S1. Search strategy 2](#_Toc226656962)

[Table S2. Summarize of eligible studies 3](#_Toc226656963)

[Table S3. Objectives of MED responding to OH JPA 4](#_Toc226656964)

[Figure S1. Main gaps analysis of eligible studies 5](#_Toc226656965)

[Figure S2. The developed operational tool for minimum essential dataset 6](#_Toc226656966)

[References 7](#_Toc226656967)

# Table S1. Search strategy

| **PubMed** | |
| --- | --- |
| 1 | ("minimal dataset"[Title/Abstract]) OR ("minimal data set"[Title/Abstract]) OR ("minimal database"[Title/Abstract]) OR ("minimum dataset"[Title/Abstract]) OR ("minimum data set"[Title/Abstract]) OR ("minimum database"[Title/Abstract]) OR ("essential dataset"[Title/Abstract]) OR ("essential data set"[Title/Abstract]) OR ("essential database"[Title/Abstract]) OR ("core dataset"[Title/Abstract]) OR ("core data set"[Title/Abstract]) OR ("core database"[Title/Abstract]) |
| 2 | ("surveillance"[Title/Abstract]) OR ("monitoring"[Title/Abstract]) |
| 3 | ("reporting"[Title/Abstract]) OR ("report"[Title/Abstract]) OR ("notification"[Title/Abstract]) |
| 4 | #2 OR #3 |
| 5 | #1 AND #4 |
| Results: 987 studies | |
| **Web of Science (core database)** | |
| 1 | TS=("minimal dataset") OR TS=("minimal data set") OR TS=("minimal database") OR TS=("minimum dataset") OR TS=("minimum data set") OR TS=("minimum database") OR TS=("essential dataset") OR TS=("essential data set") OR TS=("essential database") OR TS=("core dataset") OR TS=("core data set") OR TS=("core database") |
| 2 | TS=("surveillance") OR TS=("monitoring") |
| 3 | TS=("reporting") OR TS=("report") OR TS=("notification") |
| 4 | #2 OR #3 |
| 5 | #1 AND #4 |
| Results: 1094 studies | |
| **Embase (＜1966-2024)** | |
| 1 | 'minimal dataset':ab,ti OR 'minimal data set':ab,ti OR 'minimal database':ab,ti OR 'minimum dataset':ab,ti OR 'minimum data set':ab,ti OR 'minimum database':ab,ti OR 'essential dataset':ab,ti OR 'essential data set':ab,ti OR 'essential database':ab,ti OR 'core dataset':ab,ti OR 'core data set':ab,ti OR 'core database':ab,ti |
| 2 | 'surveillance':ab,ti OR 'monitoring':ab,ti |
| 3 | 'reporting':ab,ti OR 'report':ab,ti OR 'notification':ab,ti |
| 4 | #2 OR #3 |
| 5 | #1 AND #4 |
| Results: 1387 studies | |
| **Scopus** | |
| 1 | ( TITLE-ABS-KEY ( "core database" ) ) OR ( TITLE-ABS-KEY ( "core data set" ) ) OR ( TITLE-ABS-KEY ( "core dataset" ) ) OR ( TITLE-ABS-KEY ( "essential database" ) ) OR ( TITLE-ABS-KEY ( "essential data set" ) ) OR ( TITLE-ABS-KEY ( "essential dataset" ) ) OR ( TITLE-ABS-KEY ( "minimum database" ) ) OR ( TITLE-ABS-KEY ( "minimum data set" ) ) OR ( TITLE-ABS-KEY ( "minimum dataset" ) ) OR ( TITLE-ABS-KEY ( "minimal database" ) ) OR ( TITLE-ABS-KEY ( "minimal data set" ) ) OR ( TITLE-ABS-KEY ( "minimal dataset" ) ) |
| 2 | (TITLE-ABS-KEY ("surveillance") OR TITLE-ABS-KEY ("monitoring")) |
| 3 | (TITLE-ABS-KEY ("reporting") OR TITLE-ABS-KEY ("report") OR TITLE-ABS-KEY ("notification" )) |
| 4 | #2 OR #3 |
| 5 | #1 AND #4 |
| Results: 1560 studies | |

# Table S2. Summarize of eligible studies

| **Characteristic** | **Content** | **Implementation process** | **Reference** |
| --- | --- | --- | --- |
| Research to establish MED | Literature search and Delphi survey | ⅰ) Searching literature using keywords; ⅱ) Extracting elements from included literature; ⅲ) Developing a questionnaire using the selected data elements; ⅳ) Formatting expert groups; ⅴ) Expert review of data list and scoring; ⅵ) Elements of consensus are incorporated into MED. | Shafiee et al. [1]; Kazemi-Arpanahi et al. [2]; Shanbehzadeh et al. [3-5] |
|  | Data collection and expert panel | ⅰ) Searching information from multiple sources (literature, information systems, report, document…); ⅱ) Extracting elements from included literature; ⅲ) Developing a questionnaire using the selected data elements; ⅳ) Formatting expert groups; ⅴ) Expert review of data list and scoring; ⅵ) Elements of consensus are incorporated into MED. | Shanbehzadeh et al. [6]; Liu et al. [7] |
|  | Literature review, expert panel discussion sessions and pilot study | ⅰ) Assessing the information requirements; ⅱ) Identifying data elements (literature review, internet searching, request information from experts, on-site review of information systems, document review and interview of experts); ⅲ) Selecting the MED (expert panel method); ⅳ) Implementing a pilot study at hospital to test the MED; ⅴ) Evaluating and then correcting the MED. | Zarei et al. [8, 9] |
|  | Systematic review | ⅰ) Identifying research questions; ⅱ) Developing a search strategy; ⅲ) Searching literature from databases; ⅳ) Screening literature; ⅴ) Extracting data elements; ⅵ) The extracted data elements are incorporated into the MED. | Shanbehzadeh et al. [10]; Haghiri et al. [11] |
|  | Qualitative study | ⅰ) Selecting research subjects; ⅱ) Designing the questionnaire based on the common characteristics of the subjects and the purpose of the study; ⅲ) Study participants fill out the questionnaire; ⅳ) MED applicable to the study population was summarized from the results of questionnaire. | Schmidt et al. [12] |
| Review of previously established MED | Summarize previously established MED | Summarizes the subjects, findings, and scope of application of established MED, without specifically explaining the methodology and process of establishing MED. | Kloeze et al. [13]; da Costa et al. [14]; Allan et al. [15]; Rieder et al. [16]; Trotter et al. [17] |
|  | Summarize and compare previously established MED across countries | Based on the summarized characteristics, differences among MEDs are compared to provide recommendations for integrating and improving these MEDs. | Taramasco et al. [18]; Bagherian et al. [19]; Adlhoch et al. [20] |
| Recommend to establish MED | A MED is needed for the disease under study. | ˗ | Lau et al. [21]; Zhou et al. [22]; Lanata et al. [23]; Vial et al. [24]; Lowndes et al. [25]; Giesecke J [26]; Foraker et al. [27] |
|  | The content of MED needs to be added and updated. | ˗ | Rea et al. [28] |

**Notes:** MED, minimum essential dataset.

# Table S3. Objectives of MED responding to OH JPA

| **Action track of OH JPA** | **Objectives of surveillance** | **Diseases involved in established MED** | **Reference** |
| --- | --- | --- | --- |
| **Action track 1:** Enhancing One Health capacities to strengthen health systems | Recommend to improve MED through One Health approach | ˗ | Shanbehzadeh et al. [4]； Kloeze et al. [13]； Zhou et al. [22] |
| **Action track 2:** Reducing the risks from emerging and re-emerging zoonotic epidemics and pandemics | Emerging and re-emerging zoonotic diseases | Brucellosis, COVID-19, SARS, Zoonotic diseases, Influenza A (H7N9), Tuberculosis | Shafiee et al. [1]; Shanbehzadeh et al. [3]; Kazemi-Arpanahi et al. [2]; Shanbehzadeh et al. [4]; Shanbehzadeh et al. [6]; Zarei et al. [8]; Zarei et al. [9]; da Costa et al. [14]; Allan et al. [15]; Rieder et al. [16]; Lau et al. [21]; Rea et al. [28]; Foraker et al. [27] |
| **Action track 3:** Controlling and eliminating zoonotic, neglected tropical and vector-borne diseases | Zoonotic and neglected tropical diseases | Neglected tropical diseases | Shanbehzadeh et al. [4]; Zhou et al. [22] |
| **Action track 4:** Strengthening the assessment, management and communication of food safety risks | Food-borne diseases | Hepatitis E virus infection | Adlhoch et al. [20] |
| **Action track 5:** Curbing the silent pandemic of Antimicrobial Resistance (AMR) | ˗ | ˗ | ˗ |
| **Action track 6:** Integrating the Environment into One Health | Integration of environmental data elements | Zoonotic diseases | Shanbehzadeh et al. [4] |

**Notes**: MED, minimum essential dataset; OH JPA, One Health Joint Plan of Action.

# Figure S1. Main gaps analysis of eligible studies

(Each axis in the figure represents an evaluation entry, and the number of studies under each corresponding entry was counted to calculate its percentage of the total number of studies. Trak 1: Enhancing One Health capacities to strengthen health systems; Track 2: Reducing the risks from emerging and re-emerging zoonotic epidemics and pandemics; Track 3: Controlling and eliminating zoonotic, neglected tropical and vector-borne diseases; Track 4: Strengthening the assessment, management and communication of food safety risks; Track 5: Curbing the silent pandemic of Antimicrobial Resistance (AMR); Track 6: Integrating the Environment into One Health. Global: every country around the world, International: specific countries suffering the same threats. Abbreviations: OH JPA, One Health Joint Plan of Action (2022-2026))


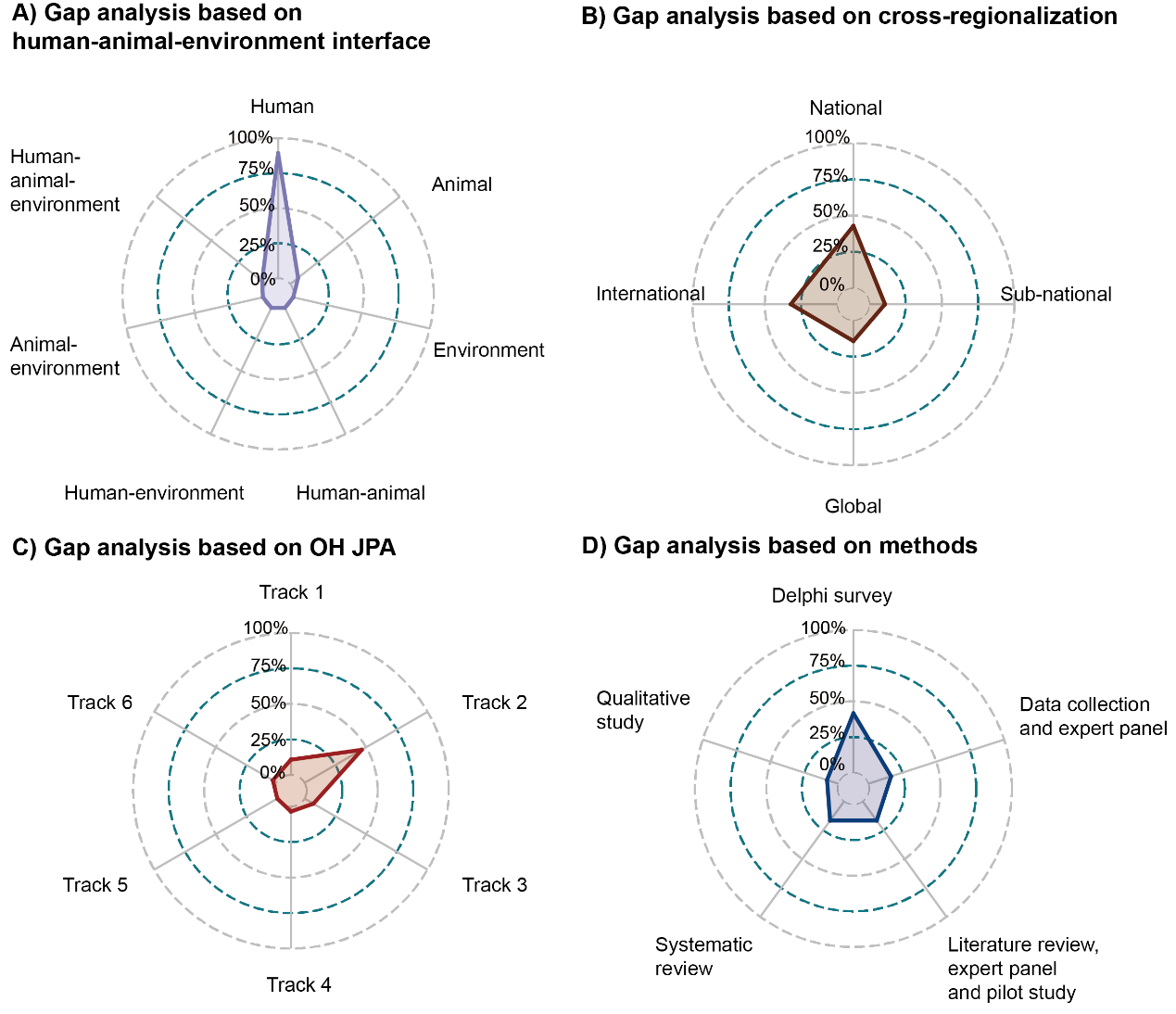


# Figure S2. The developed operational tool for minimum essential dataset

(Abbreviation: MED, minimum essential dataset)


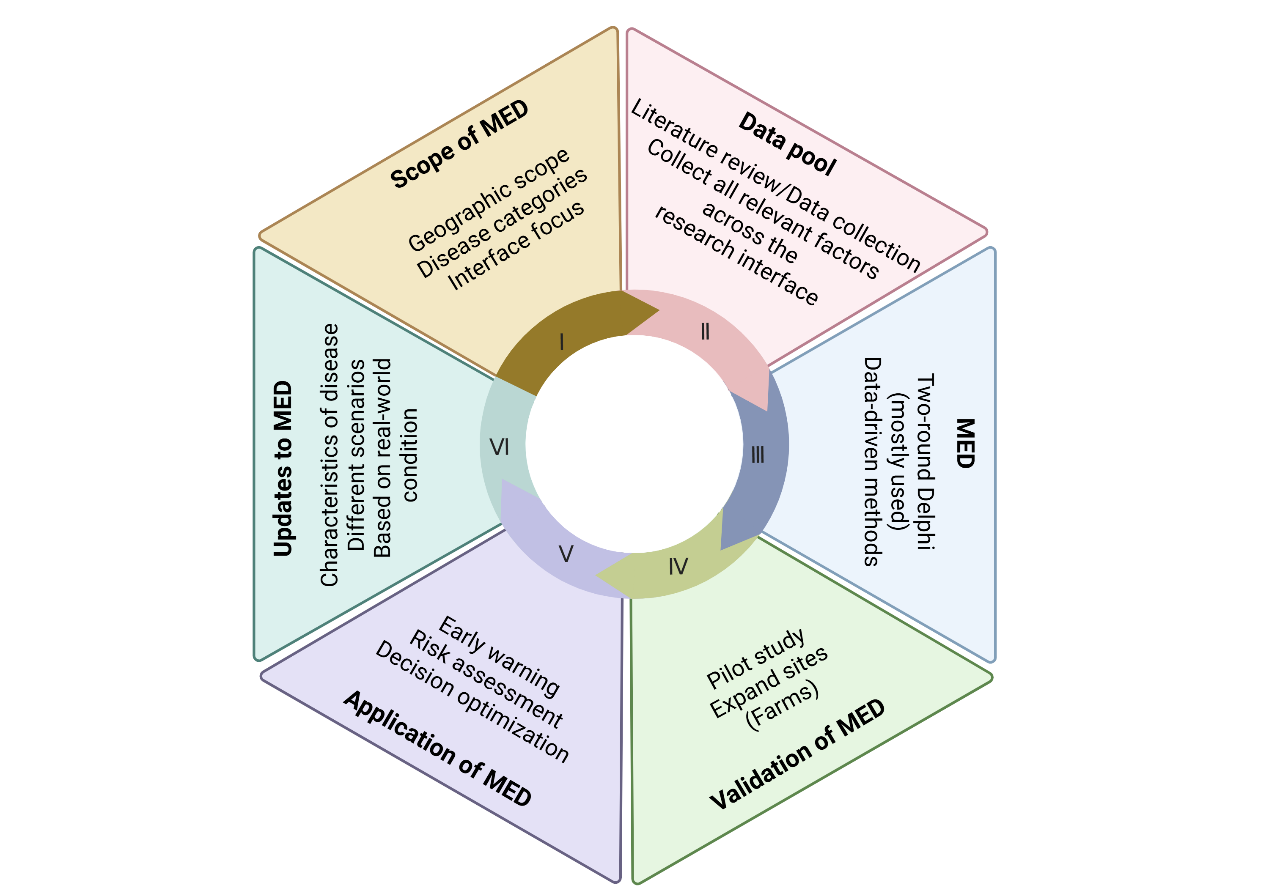


# References

1. Shafiee M, Shanbehzadeh M, Kazemi-Arpanahi H. Common data elements and features of brucellosis health information management system. Informatics in Medicine Unlocked. 2022;30. doi: 10.1016/j.imu.2022.100953.

2. Kazemi-Arpanahi H, Moulaei K, Shanbehzadeh M. Design and development of a web-based registry for Coronavirus (COVID-19) disease. Medical journal of the Islamic Republic of Iran. 2020;34:68. doi: 10.34171/mjiri.34.68.

3. Shanbehzadeh M, Kazemi-Arpanahi H, Mazhab-Jafari K, Haghiri H. Coronavirus disease 2019 (COVID-19) surveillance system: Development of COVID-19 minimum data set and interoperable reporting framework. Journal of Education and Health Promotion. 2020;9(1):203. doi: 10.4103/jehp.jehp_456_20.

4. Shanbehzadeh M, Nopour R, Kazemi-Arpanahi H. Designing a standardized framework for data integration between zoonotic diseases systems: Towards one health surveillance. Informatics in Medicine Unlocked. 2022;30. doi: 10.1016/j.imu.2022.100893.

5. Shanbehzadeh M, Kazemi-Arpanahi H, Valipour AA, Zahedi A. Notifiable diseases interoperable framework toward improving Iran public health surveillance system: lessons learned from COVID-19 pandemic. Journal of Education and Health Promotion. 2021;10(1). doi: 10.4103/jehp.jehp_1082_20.

6. Shanbehzadeh M, Kazemi-Arpanahi H. Development of minimal basic data set to report COVID-19. Medical journal of the Islamic Republic of Iran. 2020;34:111. doi: 10.34171/mjiri.34.111.

7. Liu DH, Wang X, Pan F, Xu YY, Yang P, Rao KQ. Web-based infectious disease reporting using XML forms. International Journal of Medical Informatics. 2008;77(9):630-40. doi: 10.1016/j.ijmedinf.2007.10.011.

8. Zarei J, Dastoorpoor M, Jamshidnezhad A, Cheraghi M, Sheikhtaheri A. Regional COVID-19 registry in Khuzestan, Iran: A study protocol and lessons learned from a pilot implementation. Informatics in medicine unlocked. 2021;23:100520. doi: 10.1016/j.imu.2021.100520.

9. Zarei J, Badavi M, Karandish M, Haddadzadeh Shoushtari M, Dastoorpoor M, Yousefi F, et al. A study to design minimum data set of COVID-19 registry system. BMC infectious diseases. 2021;21(1):773. doi: 10.1186/s12879-021-06507-8.

10. Shanbehzadeh M, Ahmadi M. Identification of the necessary data elements to report AIDS: a systematic review. Electronic physician. 2017;9(12):5920-31. doi: 10.19082/5920.

11. Haghiri H, Rabiei R, Hosseini A, Moghaddasi H, Asadi F. Notifiable Diseases Surveillance System with a Data Architecture Approach: a Systematic Review. Acta informatica medica : AIM : journal of the Society for Medical Informatics of Bosnia & Herzegovina : casopis Drustva za medicinsku informatiku BiH. 2019;27(4):268-77. doi: 10.5455/aim.2019.27.268-277.

12. Schmidt J-E, Tozzi AE, Rava L, Glismann S, contacts obotE-Nc. The EUVAC-NET survey: national pertussis surveillance systems in the European Union, Switzerland, Norway, and Iceland. Eurosurveillance. 2001;6(6):98-104%P 205. doi: 10.2807/esm.06.06.00205-en.

13. Kloeze H, Mukhi S, Kitching P, Lees VW, Alexandersen S. Effective Animal Health Disease Surveillance Using a Network-Enabled Approach. Transboundary and Emerging Diseases. 2010;57(6):414-9. doi: 10.1111/j.1865-1682.2010.01166.x.

14. da Costa FA, Neufeld M, Hamad M, Carlin E, Ferreira-Borges C. Response measures to COVID-19 in prisons and other detention centers. International Journal of Prisoner Health. 2021;17(3):351-8. doi: 10.1108/ijph-10-2020-0080.

15. Allan M, Lièvre M, Laurenson-Schafer H, de Barros S, Jinnai Y, Andrews S, et al. The World Health Organization COVID-19 surveillance database. International Journal for Equity in Health. 2022;21. doi: 10.1186/s12939-022-01767-5.

16. Rieder H, Watson J, Raviglione M, Forssbohm M, Migliori G, Schwoebel V, et al. Surveillance of tuberculosis in Europe. Working Group of the World Health Organization (WHO) and the European Region of the International Union Against Tuberculosis and Lung Disease (IUATLD) for uniform reporting on tuberculosis cases. European Respiratory Journal. 1996;9(5):1097-104. doi: 10.1183/09031936.96.09051097.

17. Trotter CL, Chandra M, Cano R, Larrauri A, Ramsay ME, Brehony C, et al. A surveillance network for meningococcal disease in Europe. FEMS Microbiol Rev. 2007;31(1):27-36. doi: 10.1111/j.1574-6976.2006.00060.x.

18. Taramasco C, Rimassa C. Architecture Assessment of the Chilean Epidemiological Surveillance System for Notifiable Diseases (EPIVIGILA): Qualitative Study. Jmir Formative Research. 2023;7. doi: 10.2196/34387.

19. Bagherian H, Farahbakhsh M, Rabiei R, Moghaddasi H, Asadi F. National Communicable Disease Surveillance System: A review on Information and Organizational Structures in Developed Countries. Acta informatica medica : AIM : journal of the Society for Medical Informatics of Bosnia & Herzegovina : casopis Drustva za medicinsku informatiku BiH. 2017;25(4):271-6. doi: 10.5455/aim.2017.25.271-276.

20. Adlhoch C, Mand'áková Z, Ethelberg S, Epstein J, Rimhanen-Finne R, Figoni J, et al. Standardising surveillance of hepatitis E virus infection in the EU/EEA: A review of national practices and suggestions for the way forward. Journal of Clinical Virology. 2019;120:63-7. doi: 10.1016/j.jcv.2019.09.005.

21. Lau EHY, Zheng J, Tsang TK, Liao Q, Lewis B, Brownstein JS, et al. Accuracy of epidemiological inferences based on publicly available information: Retrospective comparative analysis of line lists of human cases infected with influenza A(H7N9) in China. BMC Medicine. 2014;12(1). doi: 10.1186/1741-7015-12-88.

22. Zhou XN, Bergquist R, Tanner M. Elimination of tropical disease through surveillance and response. Infectious Diseases of Poverty. 2013;2. doi: 10.1186/2049-9957-2-1.

23. Lanata CF, Rudan I, Boschi-Pinto C, Tomaskovic L, Cherian T, Weber M, et al. Methodological and quality issues in epidemiological studies of acute lower respiratory infections in children in developing countries. International Journal of Epidemiology. 2004;33(6):1362-72. doi: 10.1093/ije/dyh229.

24. Vial F, Berezowski J. A practical approach to designing syndromic surveillance systems for livestock and poultry. Preventive Veterinary Medicine. 2015;120(1):27-38. doi: 10.1016/j.prevetmed.2014.11.015.

25. Lowndes CM, Fenton KA. Surveillance systems for STIs in the European Union: Facing a changing epidemiology. Sexually Transmitted Infections. 2004;80(4):264-71. doi: 10.1136/sti.2004.010389.

26. Giesecke J. Surveillance of infectious diseases in the European Union. Lancet. 1996;348(9041):1534. doi: 10.1016/s0140-6736(05)66167-3.

27. Foraker RE, Lai AM, Kannampallil TG, Woeltje KF, Trolard AM, Payne PRO. Transmission dynamics: Data sharing in the COVID-19 era. Learn Health Syst. 2021;5(1):e10235. doi: 10.1002/lrh2.10235.

28. Rea E, Laflèche J, Stalker S, Guarda BK, Shapiro H, Johnson I, et al. Duration and distance of exposure are important predictors of transmission among community contacts of Ontario SARS cases. Epidemiology and Infection. 2007;135(6):914-21. doi: 10.1017/S0950268806007771.
